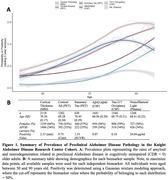# Preclinical Alzheimer Disease pathology in studies of memory and aging

**DOI:** 10.1002/alz.093516

**Published:** 2025-01-09

**Authors:** Nicole S. McKay, Peter R Millar, Nicolas R. Barthélemy, Tammie L.S. Benzinger, John C. Morris, Randall J. Bateman, Suzanne E. Schindler, Brian A. Gordon

**Affiliations:** ^1^ Washington University in St. Louis School of Medicine, St. Louis, MO USA; ^2^ Washington University in St. Louis, St. Louis, MO USA

## Abstract

**Background:**

Preclinical Alzheimer disease (AD) describes a period prior to symptom onset during which pathology begins to accumulate. Recent development of neuroimaging‐ and biofluid‐ measures of AD pathology has allowed for in vivo quantification of preclinical pathological burden. Prior work estimated that by age 85, only one third of older adults remain free of amyloid and AD‐related atrophy.

**Method:**

Here, using complementary multimodal biomarkers of AD pathology, we aimed to reproduce and extend these estimates of preclinical AD prevalence in a cohort of cognitively unimpaired (Clinical Dementia Rating = 0) older adults (demographics in Figure 1). We included neuroimaging‐derived measures of amyloid and neurodegeneration (cortical amyloid, cortical thickness, summary tau), as well as biofluid‐derived measures of non‐specific AD pathology (amyloid42:40, phosphorylated‐tau217, neurofilament light). Gaussian mixture modelling defined the probability of biomarker positivity, which we examined as a function of age (Figure 1).

**Result:**

Our results reproduce and extend prior estimates of AD pathology in cognitively unimpaired older adults, confirming in our independent sample that AD pathology is present in cognitively unimpaired individuals. Cerebrospinal fluid‐derived measures of amyloid peaked at a rate close to 60%, but all measures of amyloid and neurodegeneration were observed to rise to be present in at least 40% of cognitively unimpaired older adults. Unsurprisingly, tau was observed at the lowest rate, potentially reflecting its tight associated with cognitive impairment.

**Conclusion:**

We have replicated and extended prior reports of large proportions of older adults being afflicted by silent, preclinical, AD pathology. Rates of preclinical Alzheimer Disease pathology is important to estimate in large older adult cohorts so that these silent pathologies do not bias estimates of healthy aging. However, it is important to note that cohorts examining sporadic AD recruit individuals with family histories of AD and therefore have higher rates than average populations of apolipoprotein‐variant carriers, which may influence the rates of AD pathology. Characterizing the prevalence of preclinical AD is critical for understanding the pathophysiology of AD, may inform the design of clinical trials or interventions, and may also provide important context for furthering our understanding of cognition across older adulthood.